# Podocyte *VEGF-A* Knockdown Induces Diffuse Glomerulosclerosis in Diabetic and in *eNOS* Knockout Mice

**DOI:** 10.3389/fphar.2021.788886

**Published:** 2022-02-23

**Authors:** Delma Veron, Pardeep K. Aggarwal, Qi Li, Gilbert Moeckel, Michael Kashgarian, Alda Tufro

**Affiliations:** ^1^ Department of Pediatrics, Yale University School of Medicine, Malvern, PA, United States; ^2^ Department of Pathology, Yale University School of Medicine, New Haven, CT, United States; ^3^ Department of Cell and Molecular Physiology, Yale University School of Medicine, New Haven, CT, United States

**Keywords:** diabetic kidney disease, *VEGF* knockdown, diffuse glomerulosclerosis, S-nitrosylation, β3-integrin, laminin, GSNOR

## Abstract

Vascular endothelial growth factor-a (VEGF-A) and nitric oxide (NO) are essential for glomerular filtration barrier homeostasis, and are dysregulated in diabetic kidney disease (DKD). While NO availability is consistently low in diabetes, both high and low VEGF-A have been reported in patients with DKD. Here we examined the effect of inducible podocyte *VEGF-A* knockdown (*VEGF^KD^*) in diabetic mice and in endothelial nitric oxide synthase knockout mice (*eNOS^−/−^*). Diabetes was induced with streptozotocin using the Animal Models of Diabetic Complications Consortium (AMDCC) protocol. Induction of podocyte *VEGF^KD^* led to diffuse glomerulosclerosis, foot process effacement, and GBM thickening in both diabetic mice with intact *eNOS* and in non-diabetic *eNOS^−/−^:VEGF^KD^* mice. *VEGF^KD^* diabetic mice developed mild proteinuria and maintained normal glomerular filtration rate (GFR), associated with extremely high NO and thiol urinary excretion. In *eNOS^−/−^:VEGF^KD^* (+dox) mice severe diffuse glomerulosclerosis was associated with microaneurisms, arteriolar hyalinosis, massive proteinuria, and renal failure. Collectively, data indicate that combined podocyte *VEGF-A* and *eNOS* deficiency result in diffuse glomerulosclerosis in mice; compensatory NO and thiol generation prevents severe proteinuria and GFR loss in *VEGF^KD^* diabetic mice with intact *eNOS*, whereas *VEGF^KD^* induction in *eNOS^−/−^:VEGF^KD^* mice causes massive proteinuria and renal failure mimicking DKD in the absence of diabetes. Mechanistically, we identify *VEGF^KD^*-induced abnormal S-nitrosylation of specific proteins, including β3-integrin, laminin, and S-nitrosoglutathione reductase (GSNOR), as targetable molecular mechanisms involved in the development of advanced diffuse glomerulosclerosis and renal failure.

## Introduction

Diabetic kidney disease (DKD) is a major complication of both type 1 and type 2 diabetes that leads to renal failure, and the single most frequent cause of end-stage renal disease (ESRD) worldwide (Tuttle et al., 2014). An incomplete understanding of the molecular mechanisms that lead to DKD has precluded the development of effective treatments preventing progression to ESRD (Tufro and Veron, 2012; Reidy et al., 2014).

Vascular endothelial growth factor-A (VEGF-A) and nitric oxide (NO) are essential for glomerular filtration barrier homeostasis, and both are disregulated in diabetic nephropathy (Papapetropoulos et al., 1997; Shen et al., 1999; Hohenstein et al., 2006; Tufro and Veron, 2012). Unlike consistently low NO availability in diabetes, both high and low VEGF-A have been observed in patients with DKD (Hohenstein et al., 2006; Baelde et al., 2007; Lindenmeyer et al., 2007). We have shown that podocyte *VEGF-A* gain-of-function in diabetic mice leads to the development of Kimmelstiel-Wilson-like nodular glomerulosclerosis and massive proteinuria (Veron et al., 2011). Similar glomerular phenotype was reported in *eNOS* deficient type 1 and type 2 diabetic mouse models (Zhao et al., 2006; Nakagawa et al., 2007). Moreover, we showed that *VEGF-A* gain-of-function in *eNOS* KO mice also induces nodular glomerulosclerosis, massive proteinuria and renal failure in the absence of diabetes (Veron et al., 2014). These findings demonstrated that NO deficiency and excess VEGF-A have a synergistic deleterious effect that is necessary and sufficient for the development of nodular glomerulosclerosis, the prototypical glomerular phenotype of human advanced DKD (Veron et al., 2014). Endothelial cell or podocyte *VEGF-A* knockout causes thrombotic microangiopathy in adult mice (Lee et al., 2007; Eremina et al., 2008). Short term *VEGF-A* knockdown in podocytes induces acute renal failure and proteinuria associated with endotheliosis, mesangiolysis, and microaneurisms (Veron et al., 2012), and *VEGF-A* deletion accelerates DKD in a short term diabetes mouse model (Sivaskandarajah et al., 2012).

Here we examined the effect of podocyte *VEGF-A* knockdown (*VEGF^KD^*) in diabetic mice and in *eNOS^−/−^:VEGF^KD^* mice. We determined that in the setting of NO deficiency, caused either by diabetic milieu or *eNOS* knockout, *VEGF^KD^* results in diffuse glomerulosclerosis and proteinuria, mimicking human diabetic diffuse glomerulosclerosis of increasing severity. This phenotype is linked to the generation of NO and thiol mediated by changes in S-nitrosoglutathione reductase (GSNOR) and β3-integrin S-nitrosylation that impairs their activity.

## Methods

### Animal Models

#### A) Inducible Podocyte *VEGF^KD^: eNOS^−/−^* Mice

We generated doxycycline-inducible podocyte *VEGF^KD^* in *eNOS* KO mice (*eNOS^−/−^:VEGF^KD^*) by crossbreeding *podocin-rtTA:tet-O-siVEGF* mice (*siVEGF*) (Veron et al., 2012) with *eNOS^−/−^* (Shesely et al., 1996) (*eNOS* KO, C57BL/6j-Nos3tm1Unc; The Jackson Laboratory, Bar Harbor, ME), and we backcrossed them >8 generations to a stable FVB background. In this study *eNOS^−/−^:VEGF^KD^* mice were fed standard or doxycycline-containing chow (Harlan-Teklad) for 1 month.

#### B) *VEGF^KD^* Diabetic Mice

Diabetes was induced in 6- to 8-week-old male *siVEGF* mice (Veron et al., 2012) (herein called *VEGF^KD^*) by intraperitoneal streptozotocin (STZ) using the low dose AMDCC (Animal Models of Diabetic Complications Consortium) protocol, as previously described (Veron et al., 2011; Aggarwal et al., 2015). Random blood glucose concentration >300 mg/dl was confirmed a week after the last STZ injection and every 4 weeks along the experiment. Diabetic *VEGF^KD^* (DM-*VEGF^KD^*) and non-diabetic (non-DM-*VEGF^KD^*) mice were fed standard (−dox) or doxycycline containing chow (+dox) for 12 weeks to induce *VEGF-A* knockdown. At the end of the study 24 h urine was collected in metabolic cages; blood and kidney samples were obtained under anesthesia, as we previously described (Veron et al., 2011; Veron et al., 2012; Veron et al., 2014). All experimental protocols were approved by the Institutional Animal Care and Use Committee at Yale University School of Medicine.

### Functional Parameters

Random blood glucose was measured by glucose oxidase biosensor (OneTouch Ultra-2; LifeScan), and BP was measured under anesthesia and analyzed using PowerLab/8SP system (Chart; AD Instruments, Colorado Springs, CO, Unites States) as previously described (Veron et al., 2011; Veron et al., 2012). Plasma and urine creatinine were measured by HPLC, and glomerular filtration rate (GFR) was assessed by creatinine clearance. Albuminuria was evaluated by Coomassie blue staining and measured by ELISA (Albuwell-M, Exocell), plasma and urine VEGF-A were quantified by ELISA (R&D), NO was measured by colorimetric assay (Cayman), as previously described (Veron et al., 2014), and urine thiols (Cys and GSH) were measured by fluorometric assay (Cayman), following manufacturers’ protocol.

### Histology, Transmission Electron Microscopy, and Gene Expression

Kidneys were processed for light microscopy and TEM or frozen in isopentane, mounted in OCT (Sakura). Histology was assessed by hematoxylin/eosin and periodic acid–Schiff’s reagent (PAS) stains. TEM was performed using standard techniques, as previously described (Tsurumi et al., 1997; Veron et al., 2011). A renal pathologist (G.M.) examined all kidney samples by light and TEM, blinded to specimens’ identity (Veron et al., 2011; Veron et al., 2012; Veron et al., 2014; Aggarwal et al., 2015). Morphometric analysis was performed using point counting technique on PAS-stained sections, as previously described (Nakagawa et al., 2007). Glomerulosclerosis, mesangial expansion, mesangiolysis, endothelial injury, interstitial fibrosis, and inflammatory infiltrates were assessed using a semi-quantitative score (Véniant et al., 1994; Gross et al., 2006): 0 = none; 1 = 1–25%; 2 = 26–50%; 3 = 51–75%; 4 = 76–100% of glomerular or section areas, as appropriate (Veron et al., 2011; Veron et al., 2014). Glomerular diameters were measured in 147 ± 8 glomeruli per 5-6 mice/experimental group and glomerular volumes calculated as previously described (Reidy et al., 2009).

Immunohistochemistry (IHC) was performed in frozen kidney sections using primary antibodies against laminin, nephrin, podocin, and S-nitroso-cysteine and appropriate Cy2 and Cy3 fluorescent-tagged secondary antibodies (Jackson ImmunoResearch Laboratories), visualized by confocal microscopy (FluoView 300; Olympus), as previously described (Veron et al., 2011; Veron et al., 2012; Veron et al., 2014). Quantitation of immunofluorescent signals was performed in ≥10 glomeruli/mouse, n ≥ 4/experimental group using ImageJ software (National Institutes of Health, Bethesda, MD), as previously described (Veron et al., 2012; Aggarwal et al., 2015).

Immunoblotting was performed using the following primary antibodies: podocin (P0372, Sigma), nephrin (20R-NP002, Fitzgerald Inc.), laminin (L9393, Sigma), β3-integrin (sc-14009, Santa Cruz), VEGF receptor 2 (2479, Cell Signaling Technologies); actin (A2066. Sigma) or tubulin (Sigma) were used as a loading control. Signals were visualized by chemiluminescence, and quantified using ImageJ software (Veron et al., 2011; Veron et al., 2012; Veron et al., 2014; Aggarwal et al., 2015).

### S-Nitrosylation Assays

We evaluated GSNOR S-nitrosylation by biotin switch assay in whole kidney lysates using a S-nitrosylated protein detection kit (Cayman Chemical, Co.), as previously described (Veron et al., 2014; Li et al., 2021). Ascorbate was omitted in the labeling step to serve as negative control. We localized kidney S-nitrosylated proteins by IHC, as described (Veron et al., 2014). *In situ* Proximity Link Assays (PLA) were performed to identify specific S-nitrosylated proteins in kidney frozen sections using laminin rabbit polyclonal antibody (L9393, Sigma-Aldrich) or β3-integrin antibody (sc-14009, Santa Cruz) and S-nitrosocysteine mouse monoclonal antibody (AG Scientific) and Duolink II fluorescence protocol (Olink Bioscience, Uppsala, Sweden) (Söderberg et al., 2006), as previously described (Veron et al., 2014).

### Statistical Analyses

Data are expressed as mean ± SEM unless otherwise stated. Statistical significance (*p* < 0.05) was determined using Prism 8 software by unpaired *t* test with Welch’s correction and one-way Brown-Forsythe ANOVA to compare two or multiple experimental groups, respectively. Mann-Whitney test was used to analyze non-parametric variables.

## Results

### Podocyte *VEGF-A* Knockdown Causes Diffuse Glomerulosclerosis in Diabetic Mice

**FIGURE 1:**
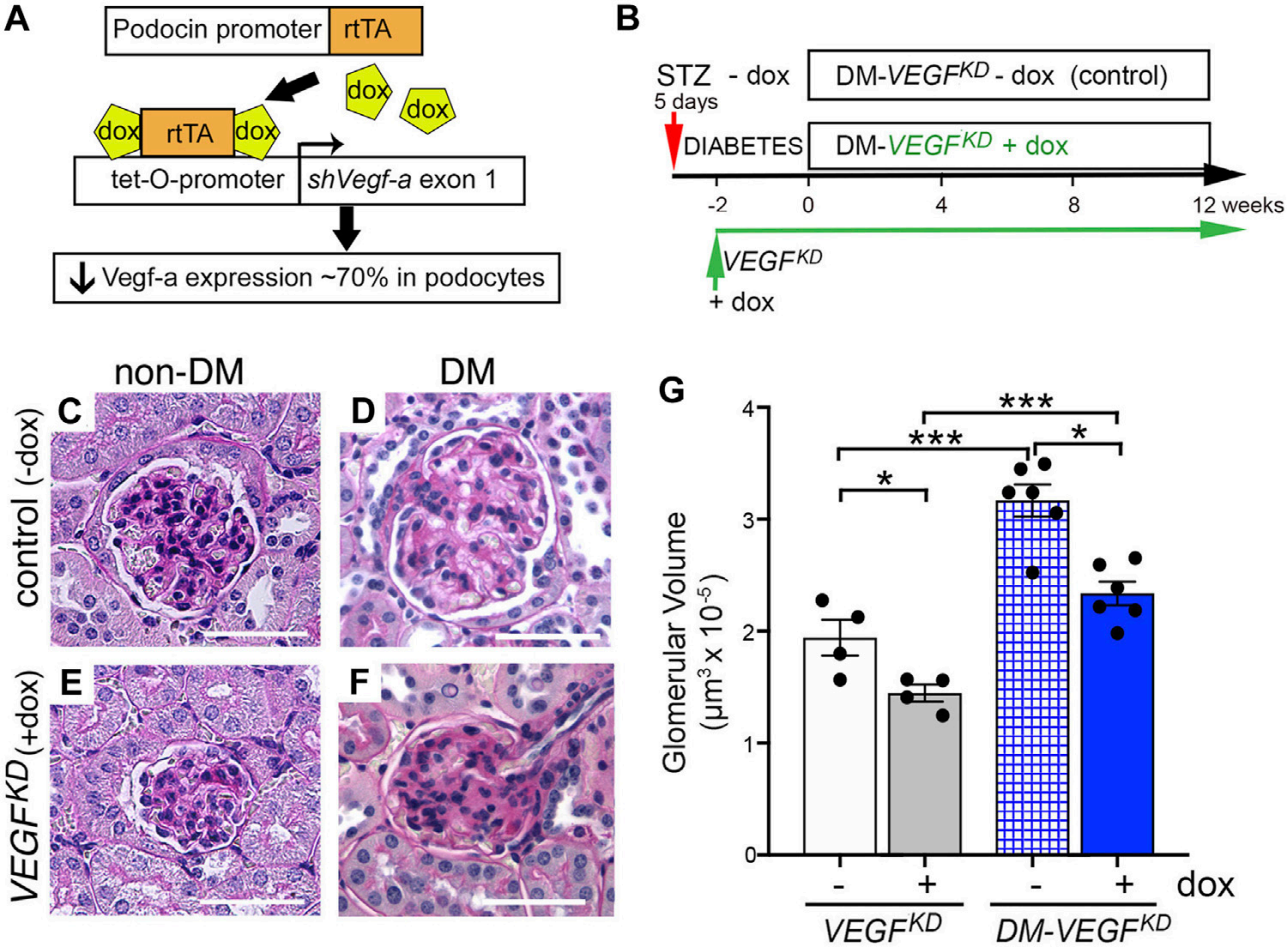
Podocyte *VEGF* knockdown (*VEGF^KD^*) prevents glomerular hypertrophy in diabetic mice. **(A)** *VEGF^KD^* Transgenic mouse line carries 4 transgenes: *Nphs2-rtTA* and *tet-0-shVEGF* that are activated by doxycycline to synthesize shRNA targeting *Vegf-a* exon 1, which inhibits expression of all *Vegf-a* isoforms in podocytes (Veron et al., 2012). **(B)** *VEGF^KD^* mice received STZ (50 mg IP, 5 daily doses) (DM-*VEGF^KD^* − dox), STZ + doxycycline (DM-*VEGF^KD^* + dox), doxycycline (*VEGF^KD^* + dox) or no treatment (*VEGF^KD^* − dox). Dox was started a week after STZ, 2 weeks later was considered time 0 (when random blood glucose was steadily elevated) for DM-*VEGF^KD^* mice; **(C)** non-diabetic control glomerulus (*VEGF^KD^* − dox) shows normal histology; **(D)** diabetic control (DM-*VEGF^KD^* − dox) glomerulus shows hypertrophy and mesangial expansion; **(E)** non-diabetic *VEGF^KD^* (+ dox) glomerulus is smaller than control (C, − dox); **(F)** diabetic *VEGF^KD^* (+ dox) glomerulus shows diffuse glomerulosclerosis and is smaller than control (D, − dox); Scale bars = 50 μm; **(G)** quantitation of glomerular size demonstrates significantly smaller glomerular volume in *VEGF^KD^* (+ dox) vs. control (−dox) glomeruli from non-diabetic (*VEGF^KD^*) and diabetic (DM-*VEGF^KD^*) mice; unpaired *t*-test with Welch’s correction was used; asterisk (*) indicates *p* < 0.05, (***) indicates *p* < 0.001; control vs. *VEGF^KD^* or non-diabetic vs. diabetic, as indicated; non-DM, non-diabetic mice, DM, diabetic mice; dox, uninduced mice; dox, doxycycline- treated mice.

Experimental design is shown in Figures 1A,B and general parameters from diabetic and non-diabetic mice are summarized in Table 1. Diabetes caused glomerulomegaly and mild mesangial expansion in uninduced (control) DM-*VEGF^KD^* − dox mice (Figures 1D,G, hatched blue bar), consistent with early diabetic kidney disease (Gundersen and Osterby, 1977; Tervaert et al., 2010). Podocyte *VEGF^KD^* induction with doxycycline for 12 weeks prevented the development of glomerular hypertrophy in DM-*VEGF^KD^* + dox mice (Figures 1F,G, blue bars), and decreased glomerular size in non-diabetic mice (Figures 1C, E, G, white and gray bars). Glomerular size in diabetic *VEGF^KD^* (+dox) mice (Figure 1F) was similar to non-diabetic control (− dox) mice (Figure 1C), as quantified in Figure 1G (white bar). However, diabetic mice with podocyte DM-*VEGF^KD^* (+dox) revealed diffuse glomerulosclerosis (Figure 1F) with limited inflammatory infiltrate and tubulo-interstitial damage. Table 2 summarizes the semi-quantitative pathology scores comparing diabetic kidneys DM-*VEGF^KD^* −dox vs. + dox.

**TABLE 1:** General parameters.

	*eNOS^−/−^:VEGF^KD^*	*eNOS^−/−^:VEGF^KD^*	DM-*VEGF^KD^*	DM-*VEGF^KD^*	*VEGF^KD^*	*VEGF^KD^*
	− dox	+ Dox	− dox	+ Dox	− dox	+ Dox
N	7–9	5	6–9	6–12	4–8	5
Age (days)	130 ± 10*	129 ± 10	198 ± 21*	190 ± 10	135 ± 4	172 ± 1****
BW(g)	25 ± 0.7	24 ± 1.3	31.5 ± 1.3	30.1 ± 1.9	31.5 ± 1***	43 ± 1.2****
KW(mg)	156 ± 12.3**	188 ± 16.8*	254 ± 12****	259 ± 18.8*	223 ± 12	257 ± 14.3
KW:BW ratio (mg/g)	6.3 ± 0.46*	8 ± 0.6*	8.2 ± 0.61	8.3 ± 0.46**	7.1 ± 0.25	6 ± 0.44
Urine volume (ml/day)	0.32 ± 0.02	0.7 ± 0.4	2.5 ± 0.2***	4.1 ± 1.7**	0.27 ± 0.03	0.2 ± 0.04
glycemia (mg/dl)	181 ± 10	165 ± 19	555 ± 29****	458 ± 42****	199 ± 14	198 ± 9
Pl plasma creatinine (mg/dl)	0.09 ± 0.003	0.17 ± 0.033*	0.11 ± 0.022	0.06 ± 0.008**	0.09 ± 0.011	0.09 ± 0.002*

Age: (Welch’s *t* test): *p = 0.0148, eNOS^−/−^:VEGF^KD^ − dox vs. DM-VEGF^KD^ − dox; *p = 0.0118, DM-VEGF^KD^ − dox vs. VEGF^KD^ − dox; ****p < 0.0001, VEGF^KD^ − dox vs. + dox. BW: body weight (Welch’s *t* test): ****p < 0.0001, VEGF^KD^ − dox vs. + dox; ****p < 0.0001, VEGF^KD^ + dox vs. DM-VEGF^KD^ + dox; ***p = 0.0001, VEGF^KD^ − dox vs. eNOS^−/−^:VEGF^KD^ − dox; ****p < 0.0001 , VEGF^KD^ + dox vs. eNOS^−/−^:VEGF^KD^ + dox. KW: kidney weight (Welch’s *t* test): **p = 0.0019, eNOS^−/−^:VEGF^KD^ − dox vs. VEGF^KD^ − dox; *p = 0.0151, eNOS^−/−^:VEGF^KD^ + dox vs. VEGF^KD^ + dox; ****p < 0.0001, DM-VEGF^KD^ − dox vs. eNOS^−/−^:VEGF^KD^ − dox; *p = 0.0153, DM-VEGF^KD^ + dox vs. eNOS^−/−^:VEGF^KD^ + dox. KW:BW ratio (Welch’s *t* test): *p = 0.03, eNOS^−/−^:VEGF^KD^ − dox vs. + dox (Mann-Whitney test); *p = 0.03, eNOS^−/−^:VEGF^KD^ + dox vs. VEGF^KD^ + dox; *p = 0.0272, eNOS^−/−^:VEGF^KD^ − dox vs. DM-VEGF^KD^ − dox;**p = 0.003, DM-VEGF^KD^ + dox vs. VEGF^KD^ + dox. Urine volume: *p < 0.025 (all groups Brown-Forsythe ANOVA test); ****p < 0.0001, DM-VEGF^KD^ vs. VEGF^KD^ − dox; **p = 0.0016, DM-VEGF^KD^ + dox vs. VEGF^KD^ + dox. Glycemia: ****P=<0.0001 DM-VEGF^KD^ vs. all non-diabetic mice (Brown-Forsythe ANOVA test). plasma Creatinine: *p = 0.01, eNOS^−/−^:VEGF^KD^ − dox vs. +dox; **p = 0.0043, DM-VEGF^KD^ + dox vs. eNOS^−/−^: VEGF^KD^ + dox mice; *p = 0.0159, VEGF^KD^ + dox vs. eNOS^−/−^: VEGF^KD^ + dox (Mann-Whitney test). All other comparisons within and between experimental groups were non significant.

**TABLE 2:** Pathology score.

Pathology Score	Endothelial injury	Mesangial sclerosis	Mesangiolysis	Inflammatory infiltrate	Interstitial fibrosis

*eNOS^−/−^:VEGF^KD^* − dox	0.6 ± 0.25	1 ± 0.32	0 ± 0	0 ± 0	0.8 ± 0.2
*eNOS^−/−^:VEGF^KD^* + dox	3 ± 0.4^a^	3.4 ± 0.4^a^	1.6 ± 0.61^a^	1 ± 0.4	1.6 ± 0.22^a^
DM-*VEGF^KD^* − dox	0 ± 0	0 ± 0	0 ± 0	1 ± 0	0 ± 0
DM-*VEGF^KD^* + dox	0 ± 0	1 ± 0	0 ± 0	1 ± 0	0 ± 0

Semi-quantitative score: 0 = none; 1 = 1–25%; 2 = 26–50%; 3 = 51–75%;4 = 76–100% of glomerular or kidney section areas, as appropriate (Véniant et al., 1994; Gross et al., 2006; Veron et al., 2011).

a Indicates *p* < 0.005 compared to eNOS^−/−^:VEGF^KD^ − dox.

### Podocyte *VEGF-A* Knockdown Causes Severe Diffuse Glomerulosclerosis in *eNOS^−/−^:VEGF^KD^* Mice

**FIGURE 2:**
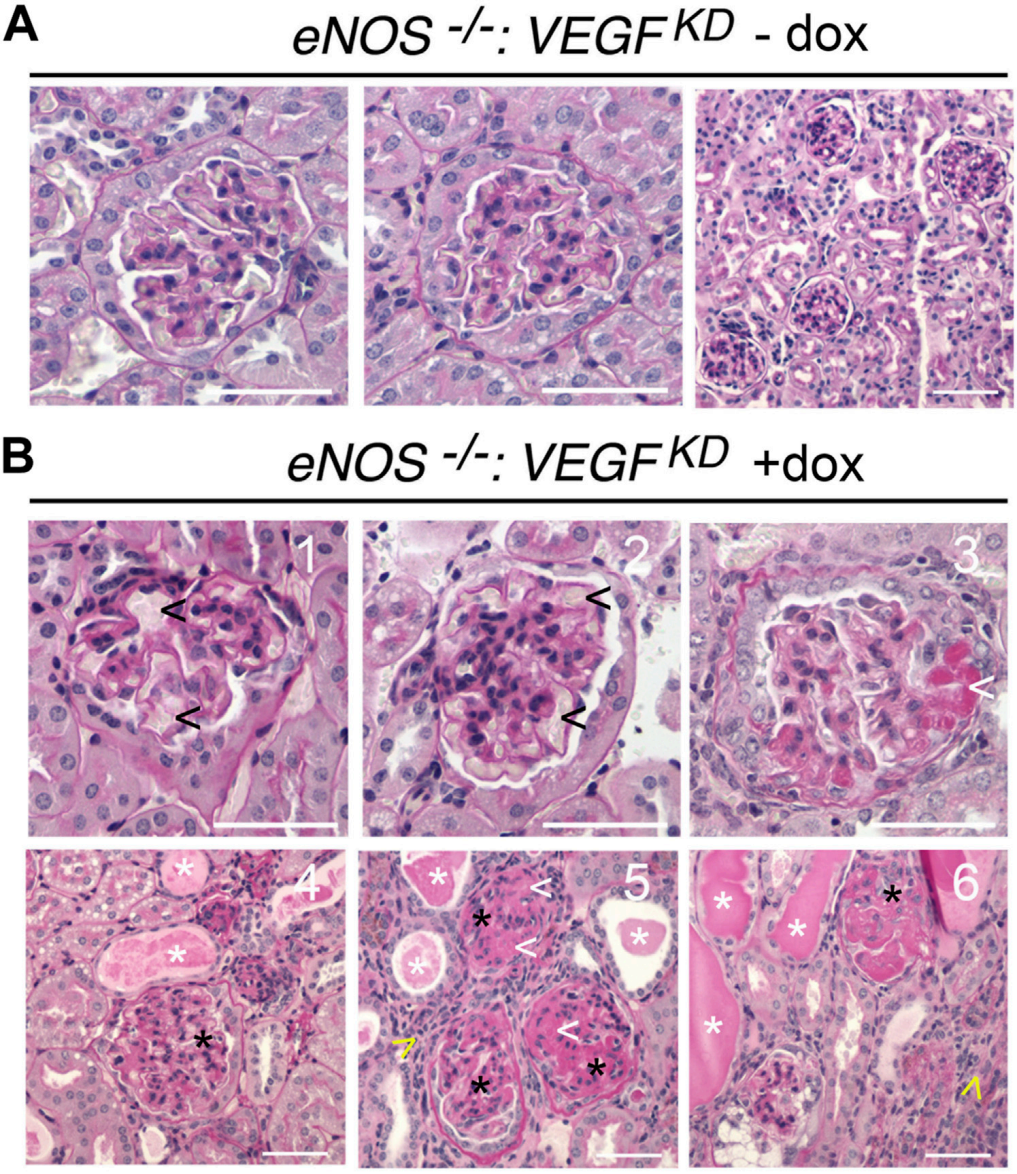
Histology of *eNOS ^−/−^:VEGF^KD^* kidneys reveals diffuse glomerulosclerosis and mimics advanced DKD: PAS stain representative images: **(A)** *eNOS^−/−^:VEGF^KD^* − dox glomeruli are normal by light microscopy; **(B)** *eNOS^−/−^:VEGF^KD^* + dox glomeruli show microaneurisms (1-2, black arrowheads), mesangiolysis (3,5,6, white arrowheads), mesangial expansion (Papapetropoulos et al., 1997; Shen et al., 1999; Reidy et al., 2014; Tuttle et al., 2014), severe mesangial sclerosis (4-6, black asterisks), proteinaceous tubular casts (4-6, white asterisks) and lymphocytic infiltrates (5–6, yellow arrowheads); Scale bars = 50 μm **(A, B1-3)** and 100 μm **(B4-6)**; PAS: Periodic acid-Schiff stain.

Glomerular histology was mostly normal in uninduced *eNOS^−/−^:VEGF^KD^* (− dox) mice (Figure 2A), although mild mesangial expansion, endothelial injury, and interstitial fibrosis were observed occasionally. Induction of podocyte *VEGF^KD^* (+dox) for 4 weeks in *eNOS^−/−^:VEGF^KD^* mice caused severe diffuse glomerulosclerosis (Figure 2B). Extensive mesangiolysis, microaneurisms, and extracellular matrix expansion were observed (Figures 2B1–3), whereas no glomerular nodules were detected in PAS stained sections from *eNOS^−/−^:VEGF^KD^* (+dox) mice. Significant tubulo-interstitial damage consisting of tubular atrophy and basement membrane thickening, tubular proteinaceous casts, and interstitial lymphocytic infiltrates were also observed in *eNOS^−/−^:VEGF^KD^* (+dox) (Figures 2B4–6) but were not present in *eNOS^−/−^:VEGF^KD^* (−dox) kidneys (Figure 2A) or in diabetic DM-*VEGF^KD^* (+dox) kidneys (Figure 1F). A semi-quantitative analysis of the histological abnormalities summarized in Table 2 confirmed these observations. Although the pathology scores revealed mild endothelial injury, mesangial sclerosis, and interstitial fibrosis in *eNOS^−/−^:VEGF^KD^* (−dox) kidneys, the severity and extension of the changes observed in *eNOS^−/−^:VEGF^KD^* (+dox) kidneys were obvious as demonstrated by highly significant score differences in all parameters.

### Podocyte *VEGF-A* Knockdown Causes Ultrastructural Glomerular Changes in Diabetic and in *eNOS^−/−^:VEGF^KD^* Mice

**FIGURE 3:**
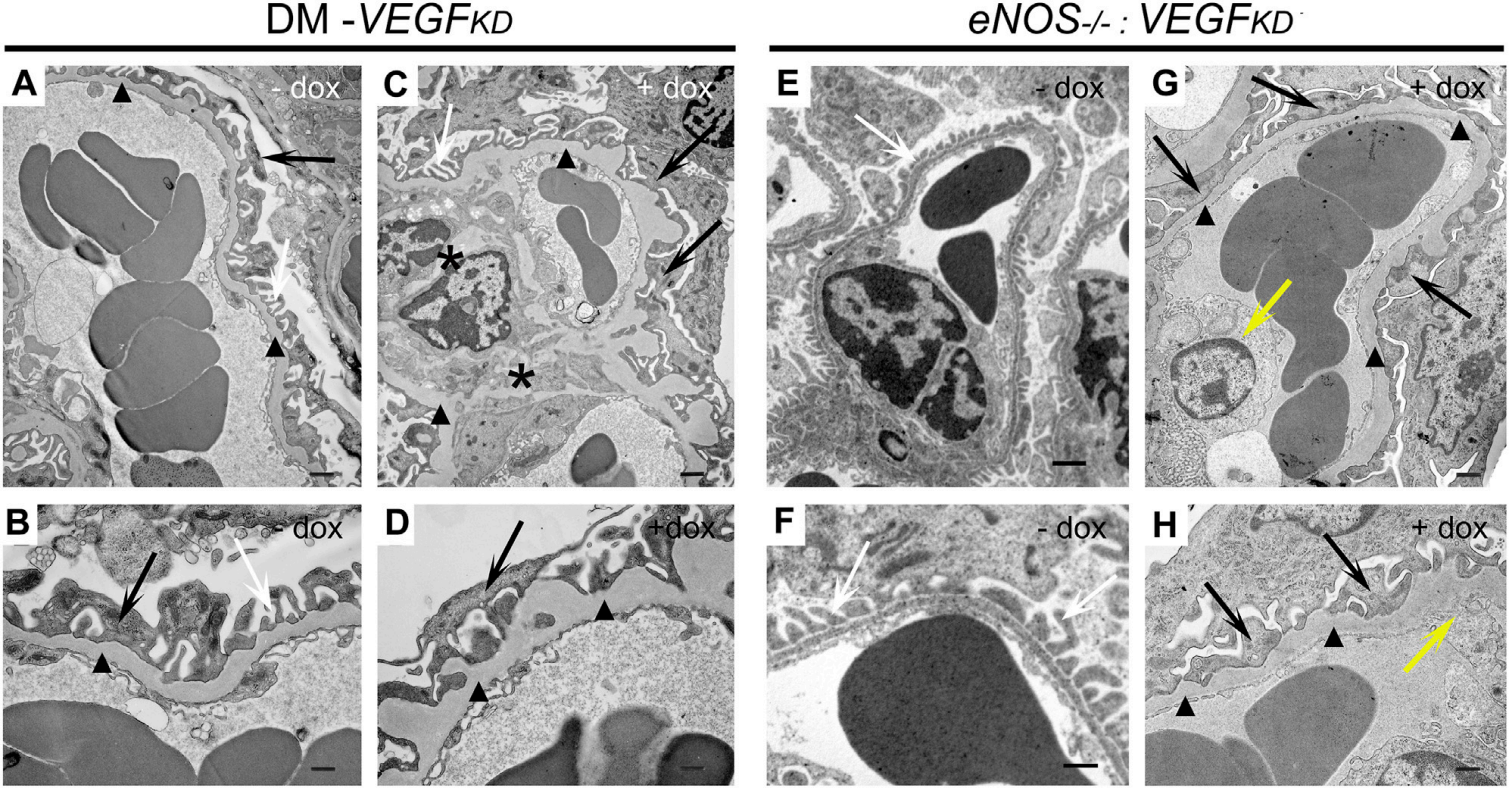
Effect of *VEGF^KD^* on glomerular ultrastructure of diabetic and *eNOS^−/−^: VEGF^KD^* kidneys. Representative TEM images: **(A,B)** diabetic control (DM-*VEGF^KD^* (− dox) glomerular capillary loop shows GBM thickening (black arrowheads) and partial foot process effacement (FPE: black arrows, normal FP: white arrows); **(C,D)** diabetic *VEGF^KD^* (+ dox) glomerulus shows mesangial sclerosis (black asterisks), extensive FPE (black arrows) and GBM thickening (black arrowheads); **(E,F)** *eNOS^−/−^:VEGF^KD^* (−dox) glomerulus shows preserved filtration barrier ultrastructure; **(G,H)** *eNOS^−/−^:VEGF^KD^* (+dox) glomerulus shows massive FPE (black arrows), GBM thickening (black arrowheads) and endotheliosis (yellow arrows). Scale bars: 1 μm in top images **(A,C,E,G)**; 500 nm in bottom images **(B,D,F,H)**; + dox:*VEGF^KD^* induction with doxycycline.

TEM revealed focal foot process effacement, mesangial expansion, and GBM thickening in all diabetic mice (Figures 3A–D), whereas mesangial sclerosis was more extensive in glomeruli from diabetic DM-*VEGF^KD^* (+dox) mice (Figure 3C). *eNOS^−/−^:VEGF^KD^* (−dox) kidneys showed normal glomeruliar filtration barrier ultrastructure (Figures 3E,F). In contrast, *eNOS^−/−^:VEGF^KD^* mice with podocyte *VEGF^KD^* (+dox) revealed extensive foot process effacement, GBM thickening, severe mesangial sclerosis, and endotheliosis (Figures 3G,H), a more severe phenotype than that observed in diabetic DM-*VEGF^KD^* (+dox) kidneys (Figures 3C,D).

### Podocyte *VEGF-A* Knockdown Causes Nephrin Downregulation in *eNOS^−/−^:VEGF^KD^* and Diabetic Mice

**FIGURE 4:**
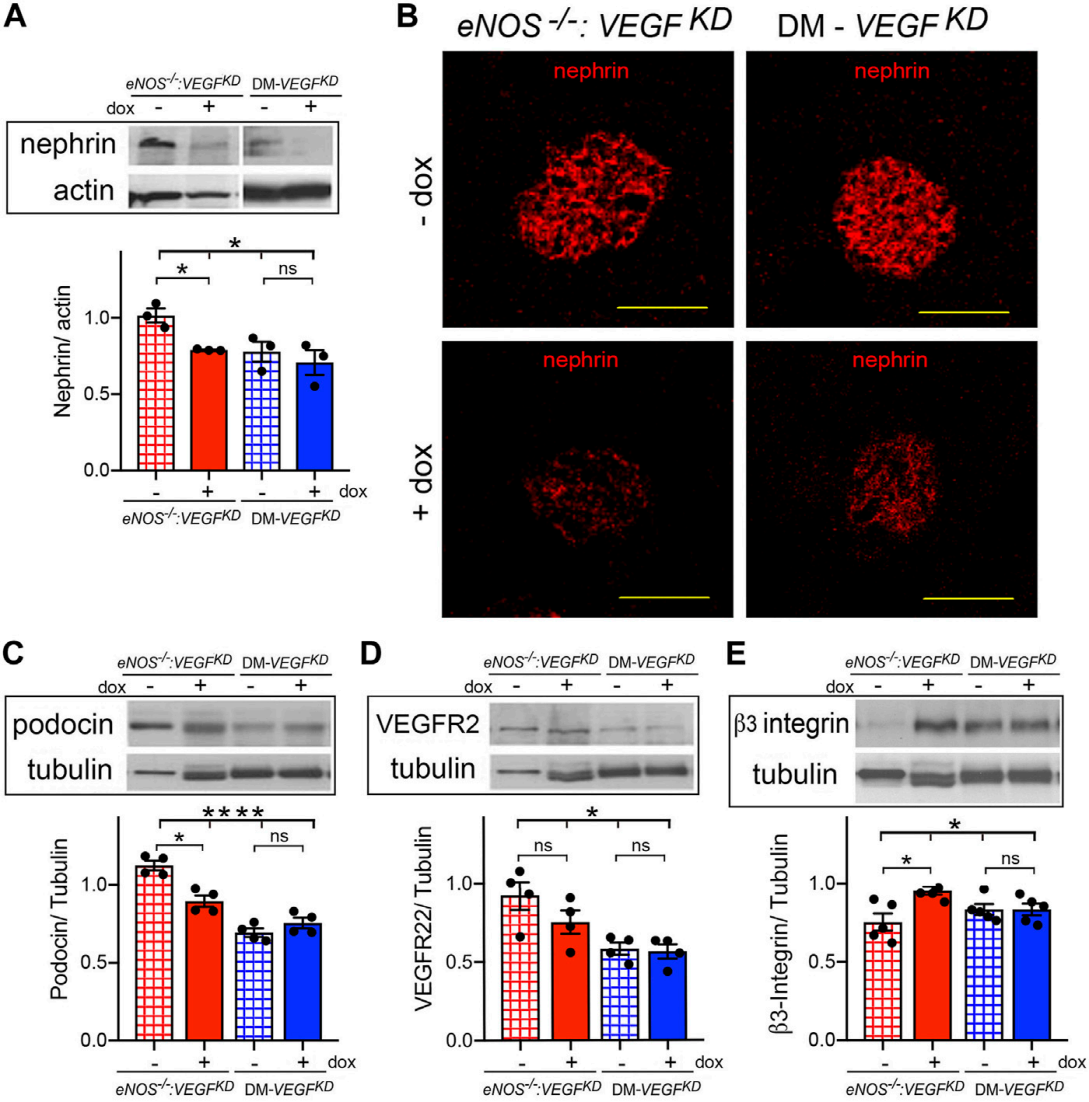
Podocyte *VEGF^KD^* downregulates nephrin in diabetic and *eNOS^−/−^:VEGF^KD^* mice: **(A)** WB: show nephrin downregulation in *eNOS^−/−^:VEGF^KD^* + dox and diabetic kidneys (Brown-Forsythe ANOVA, *p* = 0.047), no significant difference was detected between DM-*VEGF^KD^* − dox and + dox (Welch’s *t*-test) ; **(B)** IHC: nephin IF signals are clearly decreased in glomeruli from *eNOS^−/−^:VEGF^KD^* (+dox) and DM-*VEGF^KD^* (+dox) kidneys; **(C)** WB: podocin decreased in *eNOS^−/−^:VEGF^KD^* (+dox) and diabetic kidneys (Brown-Forsythe ANOVA, *p* = 0.0001), no significant difference was detected between DM-*VEGF^KD^* − dox and + dox (Welch’s *t*-test); **(D)** WB: VEGFR2 decreased in *eNOS ^−/−^:VEGF^KD^* (+dox) and diabetic kidneys (Brown-Forsythe ANOVA, *p* = 0.015) but differences (+dox vs. - dox) were not significant; **(E)** WB: significant β3-integrin upregulation was detected in *eNOS^−/−^:VEGF^KD^* (+dox) kidneys (Brown-Forsythe ANOVA, *p* = 0.03). Scale bars = 50 μm, + dox = *VEGF^KD^* induction with doxycycline.

Podocyte *VEGF^KD^* in *eNOS^−/−^:VEGF^KD^* (+dox) and diabetic mice resulted in nephrin downregulation, assessed by immunoblotting and immunohistochemistry (Figures 4A,B), and we detected similar changes in podocin expression by immunoblot (Figure 4C). Expression of VEGF-A receptor 2 (VEGFR2) was mildly decreased in *eNOS^−/−^:VEGF^KD^* and diabetic mice subjected to *VEGF^KD^* (+dox) (Figure 4D). Podocyte *VEGF^KD^* resulted in β3-integrin upregulation in *eNOS^−/−^:VEGF^KD^* (+dox) kidneys, whereas β3-integrin protein expression was not altered in diabetic mice with intact *eNOS* (Figure 4E). Collectively, these changes in protein expression levels suggest dysregulation of the nephrin-VEGFR2-β3-integrin pathway, which is necessary for the structural and functional integrity of the glomerular filtration barrier (Bertuccio et al., 2011; Veron et al., 2012; Aggarwal et al., 2015; Hayek et al., 2017).

### Podocyte *VEGF-A* Knockdown Induces Massive Proteinuria and Renal Failure in *eNOS^−/−^:VEGF^KD^* Mice, but Does Not Accentuate Proteinuria in Diabetic Mice

**FIGURE 5:**
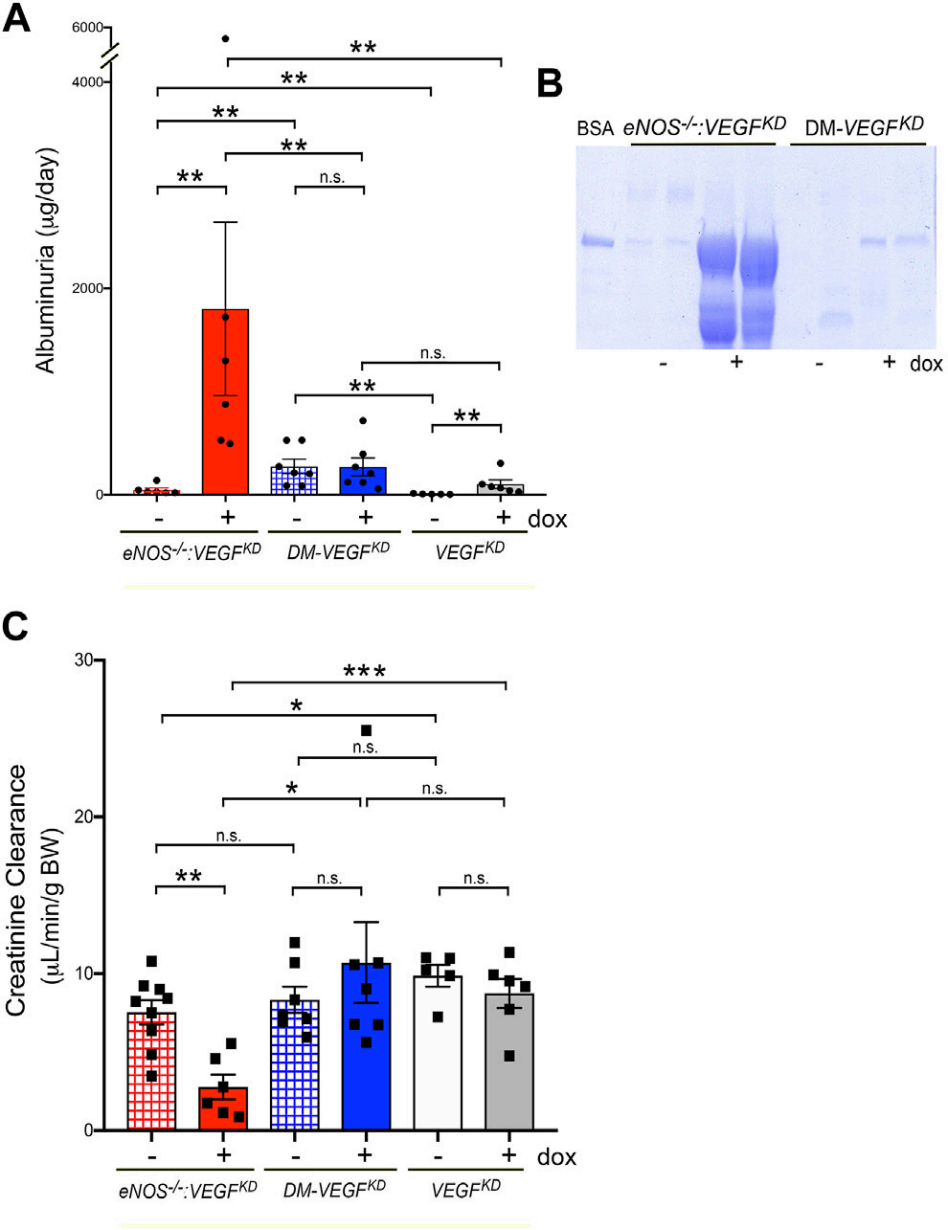
Podocyte *VEGF^KD^* causes massive proteinuria and renal failure in *eNOS^−/−^:VEGF^KD^* mice. **(A)** Induction of *VEGF^KD^* in *eNOS^−/−^:VEGF^KD^* (+dox) mice (red bar) increases albuminuria ∼30 fold higher than in control *eNOS^−/−^:VEGF^KD^* (− dox) (**, *p* = 0.0022) but does not change albuminuria in diabetic mice (DM-*VEGF^KD^* + dox, blue bar) (n.s., *p* = 0.9015); *VEGF^KD^* causes mild albuminuria in non-diabetic mice (*VEGF^KD^* + dox, gray bar) compared to controls (*VEGF^KD^* − dox, white bar) (**, *p* = 0.0043), Mann-Whitney test. **(B)** SDS PAGE/Coomassie stain shows severe albuminuria in *eNOS^−/−^:VEGF^KD^* + dox and milder albuminuria in diabetic *VEGF^KD^* + dox mice; BSA = bovine serum albumin marker, urine volume loading was normalized to creatinine. **(C)** Creatinine clearance decreases upon *VEGF^KD^* induction in *eNOS^−/−^:VEGF^KD^* + dox mice (red bar) to ∼1/3 of control *eNOS^−/−^:VEGF^KD^* − dox (***, *p* = 0.0009), but is not significantly altered in diabetic mice (DM-*VEGF^KD^* − dox and + dox, hatched/blue bars) (n.s., *p* = 0.4114) or *VEGF^KD^* in non-diabetic mice (*VEGF^KD^* − dox and + dox, white/gray bars) (n.s., *p* = 0.359) with intact eNOS; induced *eNOS^−/−^:VEGF^KD^* (+dox) mice had significantly lower Creat Cl than diabetic *VEGFKD* + dox and non-diabetic *VEGF^KD^* + dox mice (*, *p* = 0.02 and ***, *p* = 0.0007, respectively).

Induction of podocyte *VEGF^KD^* in *eNOS^−/−^:VEGF^KD^* (+dox) mice caused massive albuminuria >30-fold higher than that measured in uninduced genetically identical mice *eNOS^−/−^:VEGF^KD^* (−dox), (Figures 5A,B, red bars) and ∼15 fold higher than in *eNOS^−/−^* mice (data not shown). In contrast, mice with intact *eNOS* developed mild proteinuria when podocyte *VEGF^KD^* (+dox) was induced (Figure 5A, white/gray bars), suggesting that *eNOS* and *VEGF-A* deficiency have synergistic effect worsening proteinuria. Surprisingly, podocyte *VEGF^KD^* for 12 weeks did not increase albuminuria in diabetic (DM-*VEGF^KD^* + dox) mice (Figures 5A,B blue bars). Hypertension was not observed in non-diabetic *eNOS^−/−^: VEGF^KD^* mice (mean BP = 84 ± 2 mmHg vs. 78 ± 2 mmHg, + dox vs. −dox, pNS), as reported in *VEGF^KD^* mice with intact *eNOS* (Veron et al., 2012). Podocyte *VEGF^KD^* caused renal failure in *eNOS^−/−^:VEGF^KD^* (+dox) mice (Figure 5C, red bar), whereas it did not significantly alter creatinine clearance in mice with diabetes (DM-*VEGF^KD^*) or intact *eNOS* (*VEGF^KD^*) (Figure 5C, blue and gray bars, respectively). Taken together, these findings suggest that eNOS insufficiency and *VEGF-A* knockdown have additive pathogenic effects leading to renal failure when a compensatory NO source is not available.

### Diabetic Milieu and *eNOS^−/−^* Dysregulate *VEGF-A* and NO

**FIGURE 6:**
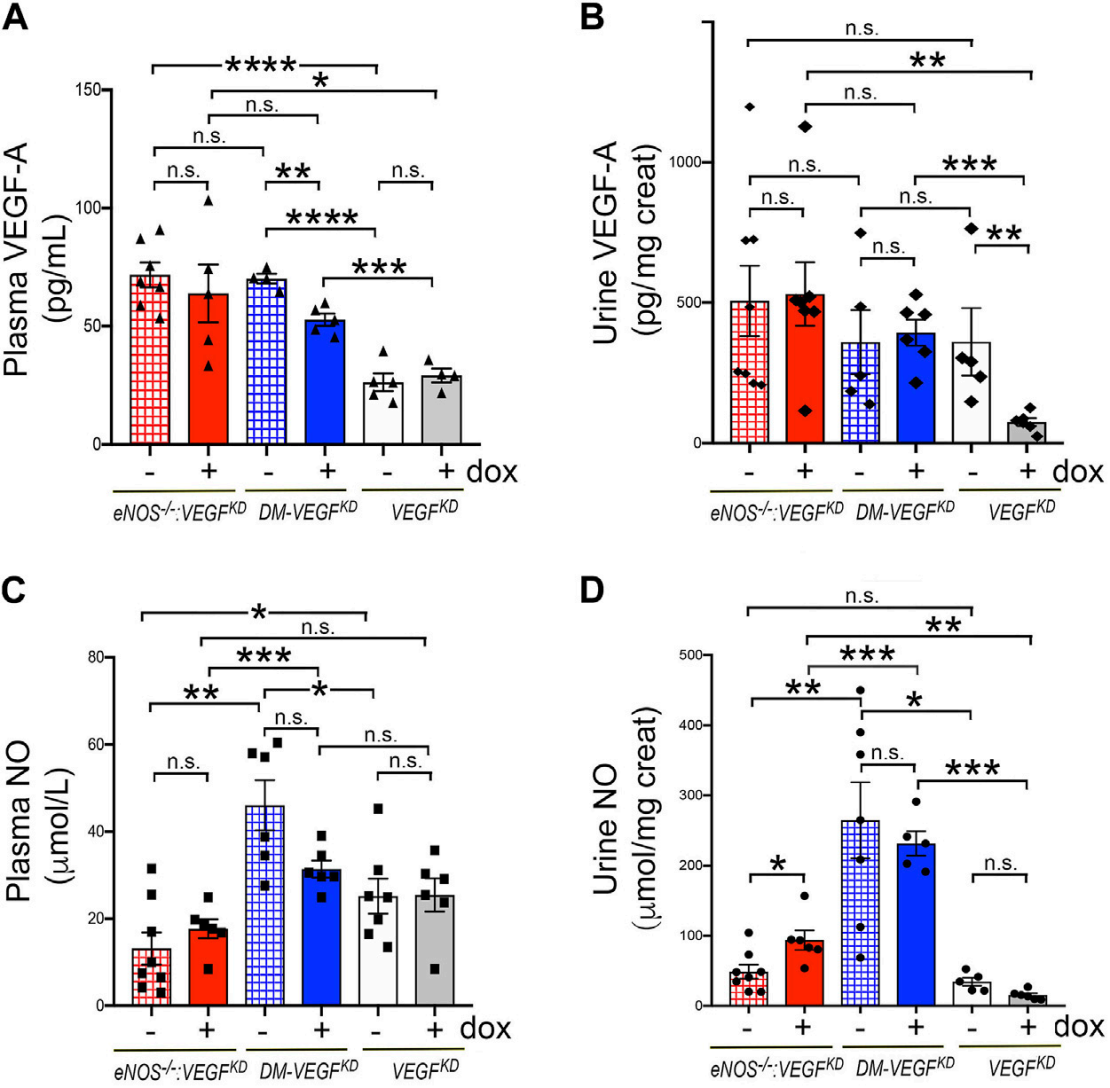
Effect of *VEGF^KD^* on circulating and urine VEGF-A and NO in diabetic and *eNOS^−/−^:VEGF^KD^* mice. **(A)** plasma VEGF-A is similarly elevated in *eNOS^−/−^:VEGF^KD^* mice (red bars) irrespectively of *VEGF^KD^*, as compared to non-diabetic *eNOS* intact mice (*VEGF^KD^* − dox, white bar) (****, *p* =<0.0001) or *VEGF^KD^* + dox mice (gray bar) (*, *p* < 0.005); in diabetic mice *VEGF^KD^* (DM-*VEGF^KD^* + dox, blue bar) significantly decreases circulating VEGF-A (**, *p* = 0.0013), but all diabetic mice have plasma VEGF-A >2-fold higher than non-diabetic mice with intact *eNOS* (*VEGF^KD^*, white/gray bars) (****, *p* < 0.0001 and ***, *p* = 0.0006). **(B)** Urine VEGF-A: podocyte *VEGF^KD^* does not alter VEGF-A excretion in *eNOS^−/−^:VEGF^KD^* (red bars) or diabetic mice (blue bars); *VEGF^KD^* significantly inhibits VEGF-A excretion in non-diabetic mice (*VEGF^KD^* + dox, gray bar) (**, *p* = 0.0043). **(C)** Plasma NO: podocyte *VEGF^KD^* (+ dox) does not significantly alter plasma NO in any experimental group; plasma NO is lower in *eNOS^−/−^:VEGF^KD^* (red bars) than diabetic (blue bars) (**, *p* = 0.001 and ***, *p* = 0.0009) and non-diabetic mice with intact *eNOS* (white bar) (*, *p* = 0.047); plasma NO is higher in diabetic (DM-*VEGF^KD^* − dox, hatched blue bar) than in non-diabetic mice (*VEGF^KD^* − dox, white bar) (*, *p* = 0.015) and *VEGF^KD^* abrogates this change (DM-*VEGF^KD^* + dox, blue bar). **(D)** Urine NO: *VEGF^KD^* increases NO excretion in *eNOS^−/−^:VEGF^KD^* + dox mice (*, *p* = 0.0272, red bar); all diabetic mice (blue bars) have several fold higher NO excretion than non-diabetic mice (white/gray bars), irrespectively of *VEGF^KD^*.

To gain insight into the availability of NO and VEGF-A systemically and at the glomerular filtration barrier we measured VEGF-A and NO in plasma and urine. We determined that plasma *VEGF-A* and urinary excretion are similarly elevated in *eNOS^−/−^:VEGF^KD^* mice, irrespective of podocyte *VEGF^KD^* (Figures 6A,B, red bars). In diabetic mice podocyte *VEGF^KD^* decreased plasma VEGF-A (Figure 6A, blue bar), which remained significantly higher (∼2-fold) than in non-diabetic mice (Figure 6A, white/gray bars). Urine VEGF-A excretion was not altered in diabetic mice, irrespective of podocyte *VEGF^KD^* (Figure 6A, blue bars). Conversely, podocyte *VEGF^KD^* in non-diabetic mice with intact eNOS significantly decreased *VEGF-A* excretion (Figure 6B, white/gray bars).

As expected, NO plasma level was low in *eNOS^−/−^:VEGF^KD^* mice (Figure 6C, red bars). In mice with intact *eNOS*, NO plasma level was higher in diabetic (blue bars) than in non-diabetic mice (white bar), but *VEGF^KD^* did not significantly decrease plasma NO in any experimental group (Figure 6C). Surprisingly, NO urinary excretion was similar in non-diabetic mice with deficient or intact *eNOS*, *VEGF^KD^* increased NO excretion two-fold in *eNOS^−/−^:VEGF^KD^* mice, whereas NO excretion increased dramatically (>6 fold) in diabetic mice, irrespective of podocyte *VEGF^KD^* (Figure 6D). No correlation was detected between VEGF-A and NO plasma levels in any experimental group, nor between VEGF-A or NO and albuminuria or creatinine clearance. These findings suggest that urinary NO excretion is not determined only by *eNOS* or *VEGF-A* and that the diabetic milieu elicits higher systemic NO and increases NO excretion in the urine, involving additional factors.

### Thiol Compensatory Mechanism in Diabetic and in *eNOS^−/−^:VEGF^KD^* Mice

**FIGURE 7:**
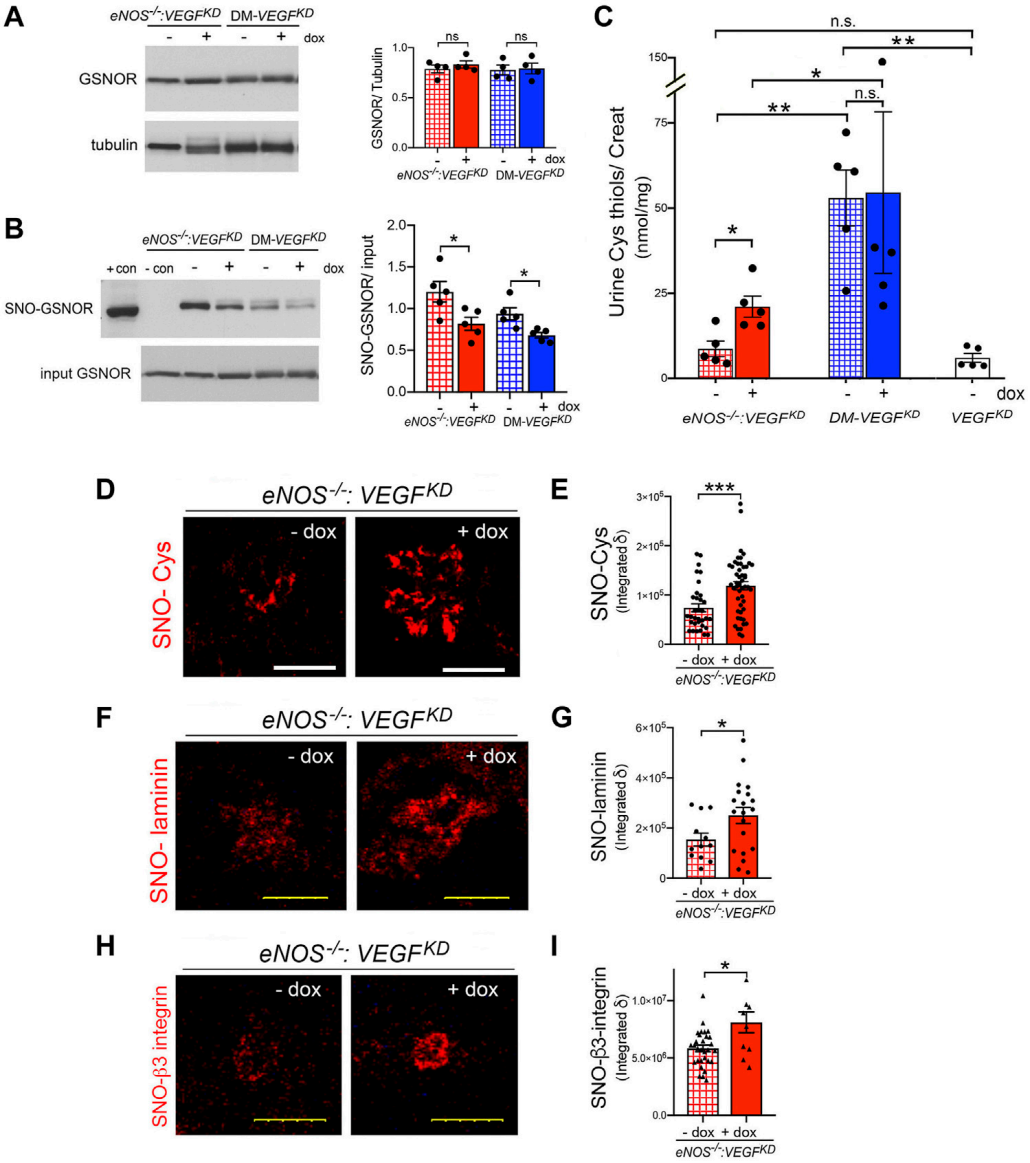
Podocyte *VEGF^KD^* induces thiol-mediated mechanisms in diabetic and *eNOS^−/−^:VEGF^KD^* mice. **(A)** WB: Kidney GSNOR expression is not altered by diabetes or *VEGF^KD^*, tubulin is shown as loading control. **(B)** GSNOR S-nitrosylation (SNO-GSNOR) detected by BST: *VEGF^KD^* (+dox) decreases SNO-GSNOR in *eNOS^−/−^:VEGF^KD^* and diabetic kidneys; podocyte and kidney lysates are used as SNO positive and negative BST controls, respectively, input shows equal loading for BST. **(C)** Urine Cys thiol excretion (normalized to creatinine): podocyte *VEGF^KD^* increases ∼2.5 fold Cys thiol excretion in *eNOS^−/−^:VEGF^KD^* + dox mice (red bar) (*, *p* = 0.013); diabetic mice (blue bars), irrespective of *VEGF^KD^*, have ∼6-fold higher Cys thiol excretion than uninduced non-diabetic mice with intact *eNOS* (*VEGF^KD^* − dox, white bar) (**, *p* = 0.004) or *eNOS^−/−^:VEGF^KD^* − dox (hatched red bar) (**, *p* = 0.004). **(D)** IHC: podocyte *VEGF^KD^* (+ dox) increases S-nitrosylation of glomerular proteins in *eNOS^−/−^:VEGF^KD^* kidneys, SNO-Cys quantification is shown in **(E)**, (****, *p* = 0.0003). **(F)** PLA: shows that podocyte *VEGF^KD^* (+ dox) increases laminin S-nitrosylation (SNO-laminin) in *eNOS^−/−^:VEGF^KD^* kidneys, SNO-laminin PLA quantification is shown in **(G)** (*, *p* = 0.025). **(H)** PLA: shows β3-integrin S-nitrosylation (SNO-β3-integrin) in *eNOS^−/−^:VEGF^KD^* glomeruli, which is increased by podocyte *VEGF^KD^* (+dox); SNO-β3-integrin quantification is shown in **(I)** (*, *p* = 0.036).

S-nitrosoglutathione (GSNO) is the major source of cellular NO not generated by NOS (Liu et al., 2001). GSNO reductase (GSNOR) deletion or decreased activity results in GSNO accumulation and promotes protein S-nitrosylation (Liu et al., 2001; Guerra et al., 2016; Stomberski et al., 2019). In turn, GSNOR activity is controlled by its S-nitrosylation (Brown-Steinke et al., 2010; Guerra et al., 2016). We determined that kidney GSNOR protein expression is not significantly altered by *eNOS^−/−^*, *VEGF^KD^* or diabetes (Figure 7A). In contrast, *VEGF^KD^* significantly decreased GSNOR S-nitrosylation in *eNOS^−/−^:VEGF^KD^* (+dox) and in DM-*VEGF^KD^* (+dox) kidneys, as detected by biotin-shift assay (BST) (Figure 7B), This SNO-GSNOR reduction could decrease GSNOR activity and lead to GSNO accumulation, providing an alternate NO source (and might mitigate glomerular damage). To assess the effect of decreased SNO-GSNOR and reductase activity, we measured thiol excretion in the urine. Cys thiol excretion was similar in non-diabetic mice with deficient (*eNOS^−/−^:VEGF^KD^* − dox) or intact eNOS (*VEGF^KD^* − dox), but increased ∼2.5 fold when *VEGF^KD^* was induced in *eNOS^−/−^:VEGF^KD^* (+ dox) mice (Figure 7C). In contrast, Cys thiol excretion was >6-fold higher in diabetic mice irrespective of *VEGF^KD^* (DM-*VEGF^KD^* −dox or + dox), than in non-diabetic mice (*eNOS^−/−^:VEGF^KD^* − dox or *VEGF^KD^* − dox) (Figure 7C). Urine GSH thiol excretion represented ∼40% of Cys-thiols in every experimental group, it was increased two-fold in *eNOS^−/−^:VEGF^KD^* (+ dox) mice and ∼5-fold in diabetic DM-*VEGF^KD^* mice than in non-diabetic mice (Supplementary Figure S1). The remarkable increase in urine thiol excretion reflects GSNO accumulation mediated by decreased SNO-GSNOR, NO generation, and protein S-nitrosylation.

### Podocyte *VEGF-A* Knockdown Increases S-Nitrosylation in *eNOS^−/−^:VEGF^KD^* Glomeruli

We examined whether *VEGF^KD^* (+dox) alters S-nitrosylation of glomerular proteins in *eNOS^−/−^:VEGF^KD^* mice. Using immunohistochemistry we determined that S-nitrosylated proteins localized to glomeruli are significantly increased in *eNOS^−/−^:VEGF^KD^* kidneys with *VEGF^KD^* (+dox) as compared to uninduced *eNOS^−/−^:VEGF^KD^* (− dox) kidneys (Figures 7D,E), indicating that podocyte *VEGF^KD^* promotes S-nitrosylation.

Specific S-nitrosylated proteins were detected *in situ* using proximity link assays (PLA). Glomerular laminin S-nitrosylation (SNO-laminin) was increased significantly in *eNOS^−/−^:VEGF^KD^* mice with *VEGF^KD^* (+dox) as compared to uninduced *eNOS^−/−^:VEGF^KD^* (− dox) mice (Figures 7F,G). Consistent with the PLA findings, immunohistochemical S-nitrosylation signals (Cys-SNO) partially co-localized with glomerular laminin were also increased in *eNOS^−/−^:VEGF^KD^* (+ dox) mice (Figure S2).

Next, we examined β3-integrin, a transmembrane protein critically involved in maintaining glomerular filtration barrier integrity (Veron et al., 2012; Hayek et al., 2017), whose activity and signaling are known to be downregulated by S-nitrosylation (Walsh et al., 2007). Using *in situ* PLA we detected β3-integrin S-nitrosylation in glomeruli (Figure 7H). Quantitation of β3-integrin PLA signals revealed that S-nitrosylated β3-integrin (SNO-β3-integrin) is increased in *eNOS^−/−^:VEGF^KD^* (+dox) kidneys as compared to uninduced *eNOS^−/−^:VEGF^KD^* (−dox) kidneys (Figure 7I). Collectively, our findings suggest that enhanced S-nitrosylation of β3-integrin and laminin may contribute to the development of diffuse glomerulosclerosis in *eNOS^−/−^:VEGF^KD^* (+dox) mice, a phenotype that mimics human advanced DKD with low VEGF.

## Discussion

This study demonstrates that in the setting of bioavailable NO deficiency, caused by diabetic milieu or by *eNOS* knockout, podocyte *VEGF-A* knockdown results in diffuse glomerulosclerosis and proteinuria of increasing severity, leading to renal failure in *eNOS^−/−^:VEGF^KD^* mice. We show that podocyte *VEGF^KD^* and *eNOS^−/−^* induce severe diffuse glomerulosclerosis in the absence of diabetic milieu. Podocyte *VEGF^KD^* in diabetic mice prevents diabetes-induced glomerulomegaly but causes diabetic diffuse glomerulosclerosis. Mechanistically, we show that compensatory local NO and thiols generation prevent severe proteinuria and GFR loss in diabetic mice with intact *eNOS*, and we identify abnormal S-nitrosylation of specific proteins, including GSNOR, laminin, and β3-integrin, as novel molecular pathways potentially involved in advanced diffuse glomerulosclerosis.

High circulating VEGF-A in diabetic mice stimulates NOS leading to NO production, protecting the integrity of the glomerular endothelium and attenuating functional abnormalities of the glomerular filtration barrier (Du et al., 2001; Nakagawa, 2008; Tufro and Veron, 2012). VEGF-A is a survival factor for all glomerular cell types and stimulates endothelial and mesangial cell proliferation (Tsurumi et al., 1997; Feliers et al., 2005; Guan et al., 2006; Lee et al., 2007), and thereby mediates glomerular hypertrophy and angiogenesis in DKD (Farquhar et al., 1959; Stout et al., 1993; Flyvbjerg et al., 2002; Veron et al., 2010; Veron et al., 2011; Tufro and Veron, 2012). Here we show that in diabetic mice podocyte *VEGF^KD^* abrogates VEGF-A-mediated glomerular hypertrophy, leading to diffuse glomerulosclerosis with modest albuminuria and normal creatinine clearance. In contrast, *eNOS^−/−^:VEGF^KD^* mice have a decreased ability to increase NO when podocyte *VEGF^KD^* is induced, despite similarly elevated circulating VEGF-A, thereby becoming more susceptible than diabetic mice to deleterious effects of local *VEGF^KD^*, resulting in mesangiolysis, extensive podocyte foot process effacement, GBM thickening, and a notably severe diffuse glomerulosclerosis phenotype reminiscent of advanced diabetic diffuse glomerulosclerosis (Farquhar et al., 1959; Tsurumi et al., 1997; Nakagawa, 2008). Moreover, *VEGF^KD^* and *eNOS* deficiency have a synergistic effect exacerbating proteinuria (>15 fold either individual genotype) and leading to renal failure, consistent with the more severe morphologic phenotype.

Previous studies demonstrated that glomerular hypertrophy and hyperfiltration occurring in diabetic mice are VEGF-A dependent (Flyvbjerg et al., 2002; Veron et al., 2010; Veron et al., 2011; Tufro and Veron, 2012), and showed that short term podocyte *VEGF* knockdown results in decreased glomerular size in non-diabetic mice (Veron et al., 2012). Here we extend this observation documenting that long term podocyte *VEGF-A* knockdown leads to significant decrease in glomerular size in non-diabetic mice and abrogates the glomerulomegaly typically observed in diabetic mice. Diabetic mice with podocyte *VEGF^KD^* developed diffuse glomerulosclerosis associated with inflammatory infiltrates and no evidence of endothelial injury or thrombotic microangiopathy (TMA). This phenotype is partially similar to that described in diabetic *VEGF-A* knockout mice (Sivaskandarajah et al., 2012), suggesting a dose effect of *VEGF-A* loss-of-function. Most mouse models of DKD show glomerular hypertrophy, mesangial, and extracellular matrix expansion (reviewed in (Brosius et al., 2009; Alpers and Hudkins, 2011). In contrast, few mouse models show advanced diabetic nodular glomerulosclerosis (Zhao et al., 2006; Nakagawa et al., 2007; Hudkins et al., 2010; Alpers and Hudkins, 2011; Veron et al., 2011; Takahashi and Harris, 2014; Aggarwal et al., 2015) or diabetic diffuse glomerulosclerosis (Alpers and Hudkins, 2011; Sivaskandarajah et al., 2012). To our knowledge, the mechanisms leading to such distinct glomerular lesions remain undefined.

*eNOS* KO mice are susceptible to developing renal failure in the setting of diabetes (Zhao et al., 2006; Nakagawa et al., 2007; Hudkins et al., 2010; Kakoki et al., 2010; Alpers and Hudkins, 2011; Yuen et al., 2012; Takahashi and Harris, 2014), reduced renal mass (Nakayama et al., 2009), and *VEGF-A* gain-of-function (Veron et al., 2014). We have previously shown that podocyte *VEGF-A* gain-of-function in *eNOS* KO mice causes massive proteinuria and renal failure (Veron et al., 2014), not unlike those described here in *eNOS^−/−^:VEGF^KD^* + dox mice, illustrating that a relatively narrow range “normal” *VEGF-A* expression and signaling at the glomerular filtration barrier are required to maintain GFR and selective permeability, as has been previously observed in other genetic and experimental models (Eremina et al., 2008; Sivaskandarajah et al., 2012; Yuen et al., 2012). Despite the similar functional consequences of podocyte *VEGF-A* gain-of-function and knockdown in *eNOS^−/−^* mice, their morphologic phenotypes are strikingly different and parallel two histologic variants of DKD described in humans: nodular or diffuse glomerulosclerosis, respectively (Farquhar et al., 1959; Stout et al., 1993). These mouse models provide the opportunity to examine the molecular pathogenic mechanisms leading to nodular or diffuse glomerulosclerosis, which are poorly understood in humans.

The Kimmelstiel-Wilson-like nodular glomerulosclerosis reported in *eNOS^−/−^* mice with excess glomerular VEGF-A is associated with decreased laminin S-nitrosylation (Veron et al., 2014). Here we demonstrate that the severe diffuse glomerulosclerosis observed in *eNOS^−/−^:VEGF^KD^* (+dox) mice is associated with increased S-nitrosylation of glomerular proteins. As opposed to loss of laminin S-nitrosylation in the setting of excess VEGF-A (Veron et al., 2014), podocyte *VEGF-A* knockdown increased laminin S-nitrosylation in *eNOS^−/−^:VEGF^KD^* (+dox) mice associates with severe diffuse glomerulosclerosis, suggesting that laminin nitrosylation might prevent the development of glomerular nodules, probably by regulating the secretion or polymerization of 521-laminin heterotrimers (Cheng et al., 1997).

**FIGURE 8:**
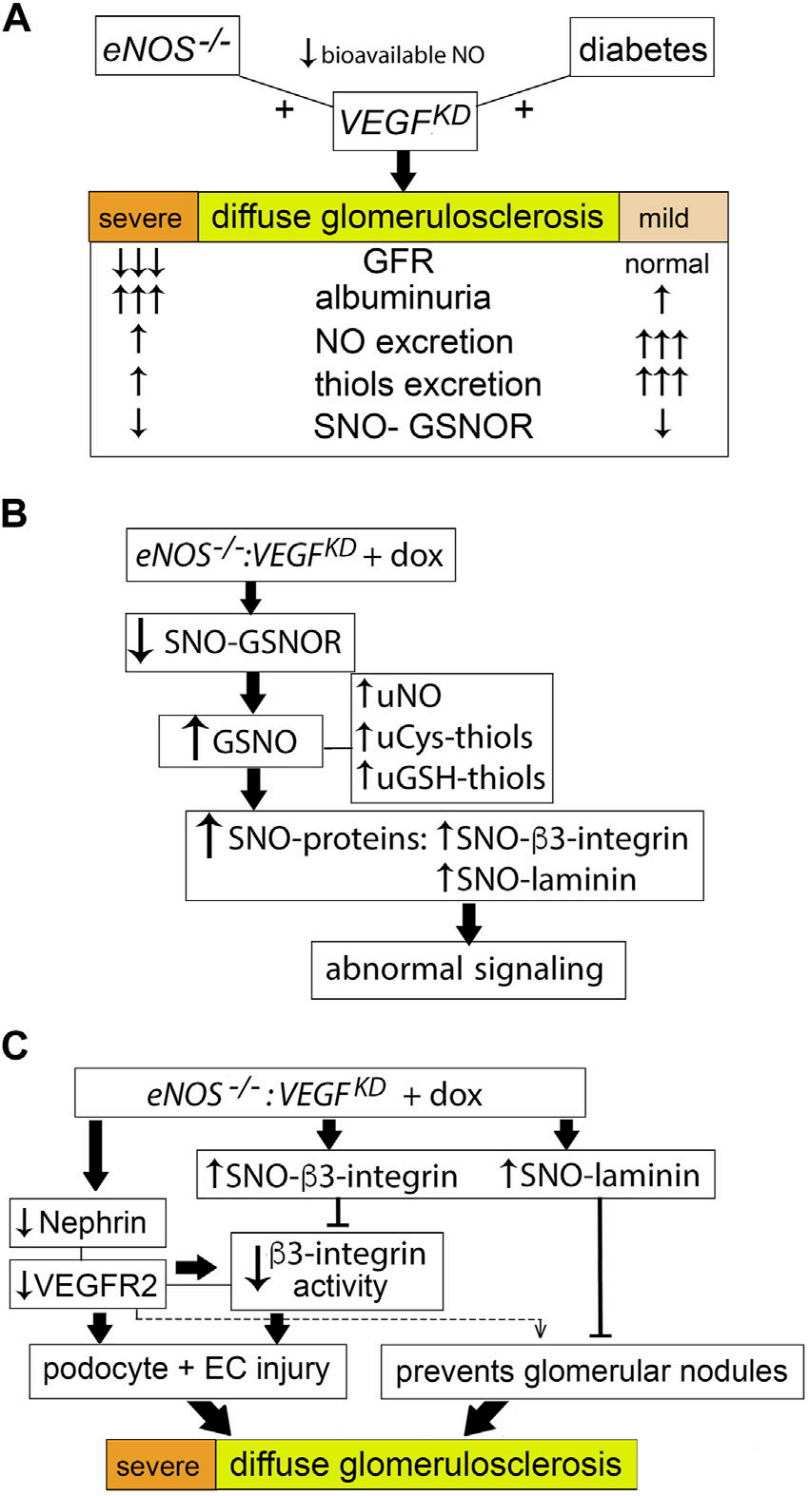
Proposed model of podocyte *VEGF^KD^* driven diffuse glomerulosclerosis in DM-*VEGF^KD^* and *eNOS:VEGF^KD^* mice. **(A)** Strong compensatory NO and thiol generation prevents GFR loss, attenuates proteinuria and diffuse glomerulosclerosis in diabetic *VEGF^KD^* mice, while limitation of this compensatory mechanism in *eNOS:VEGF^KD^* mice worsens the renal phenotype, leading to renal failure. **(B)** Reduced GSNOR S-nitrosylation increases GSNO and promotes increased S-nitrosylation of proteins, altering their signaling pathways: **(C)** decreased nephrin and VEGFR2 signaling and high SNO-β3-integrin inhibit β3-integrin activity leading to podocyte and endothelial cell injury; high SNO-laminin and low VEGFR2 signaling may contribute to the severe diffuse glomerulosclerosis described herein in *eNOS:VEGF^KD^* + dox mice.

Reversible S-nitrosylation of specific Cys residues, like Tyr phosphorylation, regulates protein-protein interactions and modulates protein function (Stamler et al., 2001; Hess and Stamler, 2012). We have recently shown that diabetic milieu dysregulates S-nitrosylation of other relevant podocyte proteins: myosin9A, RhoA and actin, activating RhoA and disrupting podocyte function in a partially reversible manner (Li et al., 2021). Thus, we examined additional S-nitrosylated proteins expressed in the kidney. GSNOR is a ubiquitous denitrosylase whose function is regulated by S-nitrosylation (Liu et al., 2001; Guerra et al., 2016; Stomberski et al., 2019). De-nitrosylation reduces GSNOR enzymatic activity in mouse cells and tissues (Brown-Steinke et al., 2010) and leads to GSNO accumulation, representing a major source of NO independent of NOS (Liu et al., 2001; Stomberski et al., 2019), although *in vitro* purified GSNOR or plant extracts decrease reductase activity upon exposure to NO donors (Guerra et al., 2016). GSNOR decreased activity was recently reported in type 2 diabetes patients and was shown to contribute to hepatic insulin resistance in an obesity mouse model (Qian et al., 2018). We determined that SNO-GSNOR was significantly decreased in *eNOS^−/−^:VEGF^KD^* (+dox) and diabetic *VEGF^KD^* (+ dox) mice. Consistent with GSNOR de-nitrosylation, we detected several fold increase in urine NO, GSH-, and Cys-thiols excretion in *eNOS^−/−^:VEGF^KD^* (+dox) and DM-*VEGF^KD^* (+dox) diabetic mice. The precise cellular origin of urine NO and thiols (ultrafiltrate, glomerular, or tubular cells) remains to be determined. We posit that GSNOR de-nitrosylation underlies the compensatory mechanism providing an alternative NO source in diabetic and *eNOS^−/−^:VEGF^KD^* (+dox) mice (Figure 8). This compensatory mechanism may support normal renal function and relatively low albuminuria in DM-*VEGF^KD^* (+dox) mice, but does not prevent the development of diffuse glomerulosclerosis. The SNO-GSNOR mediated alternate source of NO supports renal function in *eNOS^−/−^:VEGF^KD^* (− dox) mice, but it fails to do so when podocyte *VEGF^KD^* is induced (+ dox), leading to massive proteinuria and renal failure, as well as severe diffuse glomerulosclerosis, suggesting incomplete compensation or an additional *VEGF^KD^* related pathway, including iNOS activation, which we have not evaluated. GSNOR function is influenced by subcellular localization and modulated by VEGF and NOS signaling (Stomberski et al., 2019).

The novel finding that *VEGF^KD^* increases β3-integrin S-nitrosylation in *eNOS^−/−^* glomeruli might be linked to diffuse glomerulosclerosis. Laminin-521, the mature GBM laminin, binds αvβ3-integrin through interaction between α5-laminin and β3-integrin, transducing FGF and VEGF signals (Genersch et al., 2003). S-nitrosylation of β3-integrin causes conformational changes that lead to decreased integrin signaling (Walsh et al., 2007). β3-integrin S-nitrosylation in endothelial cells induces loss of integrin activity (Walsh et al., 2007; Robinson et al., 2009). We previously showed that *VEGF^KD^* decreases αvβ3-integrin activity in non-diabetic kidneys and cultured podocytes (Veron et al., 2012). Here we find that *VEGF^KD^* increases glomerular β3-integrin S-nitrosylation in *eNOS^−/−^:VEGF^KD^* (+dox) mice, likely decreasing β3-integrin signaling. Decreased β3-integrin inside-out activation disrupts nephrin-VEGFR2-β3 integrin signaling in podocytes (Bertuccio et al., 2011; Veron et al., 2012), as well as VEGFR2-β3 integrin signaling in endothelial cells (Robinson et al., 2009), leading to podocyte and endothelial injury, and eventually to diffuse glomerulosclerosis, as observed in *eNOS^−/−^:VEGF^KD^* (+ dox) mice. (Figure 8C). Whether increased S-nitrosylation impairs binding of β3-integrin and laminin-521 remains to be determined. Both decreased (Yoo et al., 2015) and increased (Maile et al., 2014) β3-integrin activity have been implicated as a mechanism of diabetic kidney disease, suggesting a context dependent role. Blockade of αvβ3-integrin activity by a monoclonal antibody improved early markers of diabetic nephropathy in pigs (Maile et al., 2014) probably by interfering with excessive VEGF-A signaling (Robinson et al., 2009; Bertuccio et al., 2011). Thus, we propose that in the setting of *VEGF^KD^* and NO deficiency, low β3-integrin activity associated with increased S-nitrosylation of β3-integrin and laminin impair growth and survival signals, resulting in severe glomerular filtration barrier disruption, leading to massive proteinuria and renal failure (Figures 8B,C).

Collectively, these findings suggest that S-nitrosylation contributes to the tight regulation of glomerular homeostasis by modulating several important signaling pathways in DKD models. Our findings support a model whereby laminin S-nitrosylation is instrumental to prevent glomerular nodule development, while GSNOR denitrosylation and increased β3-integrin S-nitrosylation lead to diffuse glomerulosclerosis in the setting of low podocyte VEGF-A.

Further studies are needed to address several limitations of this study: evaluate diabetic *eNOS^−/−^:VEGF^KD^* mice, perform a broad molecular phenotyping, confirm in cultured glomerular cell types the S-nitrosylation abnormalities identified in *eNOS^−/−^:VEGF^KD^* + dox kidneys and assess SNO-protein dysregulation in diabetic mice. Such additional studies will provide insight into how S-nitrosylation modulates several signaling pathways that are critical for glomerular homeostasis in DKD.

In summary, *VEGF^KD^* in *eNOS^−/−^:VEGF^KD^* mice causes renal failure, massive proteinuria, and severe diffuse glomerulosclerosis in the absence of diabetes. *VEGF^KD^* in diabetic mice with intact *eNOS* prevents diabetes-induced glomerulomegaly, causes diabetic diffuse glomerulosclerosis, and compensatory NO generation attenuates proteinuria and prevents GFR loss. Together, these models are reminiscent of human DKD phenotypes associated with low VEGF-A expression (Baelde et al., 2007; Lindenmeyer et al., 2007). Mechanistically, *VEGF^KD^* in *eNOS^−/−^:VEGF^KD^* mice induces increased glomerular β3-integrin S-nitrosylation, likely disrupting nephrin-VEGFR2-β3-integrin signaling (Genersch et al., 2003; Robinson et al., 2009; Bertuccio et al., 2011; Veron et al., 2012). Our observations highlight a potentially targetable novel regulatory pathway that protects the glomerular filtration barrier up to a point in mouse models that mimic human DKD.

## Data Availability

The raw data supporting the conclusion of this article will be made available by the authors, without undue reservation.
