# Supplementary figures and images for: Podocyte VEGF-A Knockdown Induces Diffuse Glomerulosclerosis in Diabetic and in eNOS Knockout Mice
Source: Front Pharmacol. 2022 Feb 23;12:788886. doi: 10.3389/fphar.2021.788886 (PMC8906751; doi:10.3389/fphar.2021.788886)

Figure S1

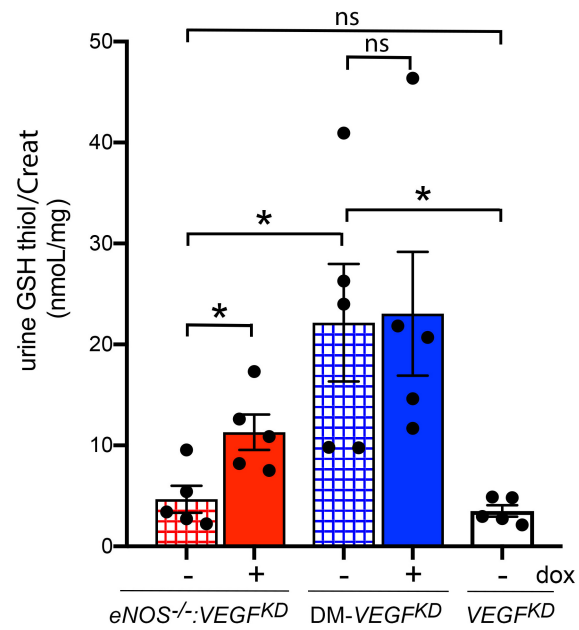

Figure S2

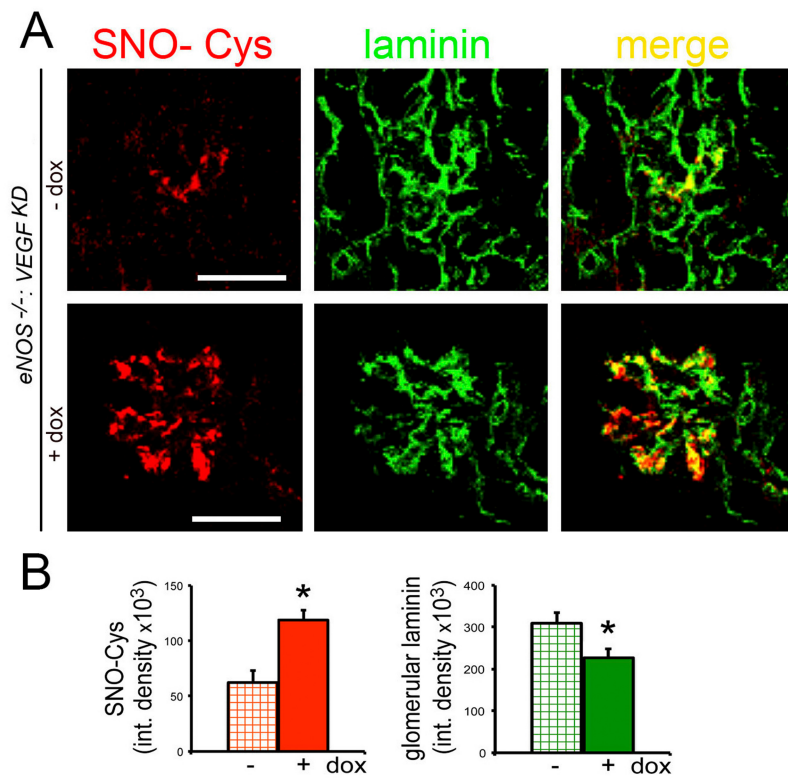

Supplement: Supplementary file 1 [file DataSheet2.PDF]
