# Supplementary material for: Podocyte VEGF-A Knockdown Induces Diffuse Glomerulosclerosis in Diabetic and in eNOS Knockout Mice
Source: Front Pharmacol. 2022 Feb 23;12:788886. doi: 10.3389/fphar.2021.788886 (PMC8906751; doi:10.3389/fphar.2021.788886)
Supplement: Supplementary file 2 [file DataSheet1.PDF]

Table 1: General parameters

	eNOS$^{-/-}$:*VEGF*^{KD} - dox	*eNOS*$^{-/-}$:*VEGF*^{KD} + dox	DM-*VEGF*^{KD} - dox	DM-*VEGF*^{KD} + dox	*VEGF*^{KD} - dox	*VEGF*^{KD} + dox
N	7-9	5	6-9	6-12	4-8	5
Age (days)	130±10*	129±10	198±21*	190±10	135±4	172±1****
BW(g)	25±0.7	24±1.3	31.5±1.3	30.1±1.9	31.5±1***	43±1.2****
KW(mg)	156±12.3**	188±16.8*	254±12****	259±18.8*	223±12	257±14.3
KW:BW ratio (mg/g)	6.3±0.46*	8±0.6*	8.2±0.61	8.3±0.46**	7.1±0.25	6±0.44
Urine volume (ml/day)	0.32±0.02	0.7±0.4	2.5±0.2***	4.1±1.7**	0.27±0.03	0.2±0.04
glycemia (mg/dl)	181±10	165±19	555±29****	458±42****	199±14	198±9
plasma creatinine (mg/dl)	0.09±0.003	0.17±0.033*	0.11±0.022	0.06±0.008**	0.09±0.011	0.09±0.002*

Age: (Welch's t test): * P=0.0148, *eNOS*$^{-/-}$:*VEGF*^{KD} – dox vs. DM-*VEGF*^{KD} – dox; * P= 0.0118, DM-*VEGF*^{KD} – dox vs. *VEGF*^{KD} – dox; **** P<0.0001, *VEGF*^{KD} – dox vs. + dox. Not significantly different (NS): *eNOS*$^{-/-}$:*VEGF*^{KD} – dox vs. + dox (P=0.9336); DM-*VEGF*^{KD} – dox vs. + dox (P=0.7143); *VEGF*^{KD} + dox vs. DM-*VEGF*^{KD} + dox.

BW: body weight (Welch's t test) : **** P<0.0001,*VEGF*^{KD} – dox vs. + dox; **** P<0.0001,*VEGF*^{KD} + dox vs. DM-*VEGF*^{KD} +dox; ***P=0.0001, *VEGF*^{KD} – dox vs. *eNOS*$^{-/-}$:*VEGF*^{KD} – dox; ****P<0.0001 , *VEGF*^{KD} + dox vs. *eNOS*$^{-/-}$:*VEGF*^{KD} + dox. NS: *eNOS*$^{-/-}$:*VEGF*^{KD} – dox vs. + dox (P=0.4082), DM-*VEGF*^{KD} – dox vs. + dox (P=0.6973).

KW: kidney weight (Welch's t test): ** P=0.0019, *eNOS*$^{-/-}$:*VEGF*^{KD} – dox vs. *VEGF*^{KD} – dox; * P=0.0151, *eNOS*$^{-/-}$:*VEGF*^{KD} + dox vs. *VEGF*^{KD} + dox; **** P<0.0001, DM-*VEGF*^{KD} – dox vs. *eNOS*$^{-/-}$:*VEGF*^{KD} – dox; * P=0.0153, DM-*VEGF*^{KD} + dox vs. *eNOS*$^{-/-}$:*VEGF*^{KD} + dox. NS: *eNOS*$^{-/-}$:*VEGF*^{KD} – dox vs. + dox (P=0.1594); DM-*VEGF*^{KD} – dox vs. + dox (P=0.8239), *VEGF*^{KD} – dox vs. + dox (P=0.1093), DM-*VEGF*^{KD} vs. *VEGF*^{KD} – dox (P=0.0908), DM-*VEGF*^{KD} vs. *eNOS*$^{-/-}$:*VEGF*^{KD} + dox (P=0.9236).

KW:BW ratio (Welch's t test): * P=0.03, *eNOS*$^{-/-}$:*VEGF*^{KD} – dox vs. + dox (Mann-Whitney test); * P=0.03, *eNOS*$^{-/-}$:*VEGF*^{KD} + dox vs. *VEGF*^{KD} +dox; * P=0.0272, *eNOS*$^{-/-}$:*VEGF*^{KD} – dox vs. DM-*VEGF*^{KD} - dox;** P=0.003, DM-*VEGF*^{KD} + dox vs. *VEGF*^{KD} + dox. NS: DM-*VEGF*^{KD} – dox vs. + dox (P=0.8576); *VEGF*^{KD} – dox vs. + dox (P=0.778); DM-*VEGF*^{KD} – dox vs. *VEGF*^{KD} – dox (P=0.116); *eNOS*$^{-/-}$:*VEGF*^{KD} - dox vs. *VEGF*^{KD} -dox (P=0.1824); *eNOS*$^{-/-}$:*VEGF*^{KD} + dox vs. DM-*VEGF*^{KD} +dox (P=0.6686).

Urine volume: * P< 0.025 (all groups Brown-Forsythe ANOVA test); ****P<0.0001, DM-*VEGF*^{KD} vs. *VEGF*^{KD} – dox; ** P=0.0016, DM-*VEGF*^{KD} + dox vs. *VEGF*^{KD} + dox. NS: *eNOS*$^{-/-}$: *VEGF*^{KD} – dox vs.+dox (P=0.1119), DM-*VEGF*^{KD} - dox vs. +dox (>0.9999), *VEGF*^{KD} – dox vs. *VEGF*^{KD} – dox (P=0.2698), *eNOS*$^{-/-}$: *VEGF*^{KD} – dox vs. *VEGF*^{KD} – dox (P=0.1150), *eNOS*$^{-/-}$: *VEGF*^{KD} +dox vs.*VEGF*^{KD} +dox (P=0.1190). ; (Mann-Whittney test).

Glycemia: ****P=<0.0001 DM-*VEGF*^{KD} vs. all non-diabetic mice (Brown-Forsythe ANOVA test); DM-*VEGF*^{KD} -dox vs. +dox mice (P=0.2901, NS).

pl Creatinine: * P=0.01, *eNOS*$^{-/-}$:*VEGF*^{KD} – dox vs. +dox; **P=0.0043, DM-*VEGF*^{KD} +dox vs. *eNOS*$^{-/-}$: *VEGF*^{KD} +dox mice; * P=0.0159, *VEGF*^{KD}+dox vs. *eNOS*$^{-/-}$: *VEGF*^{KD} +dox (Mann-Whitney test); NS (Mann-Whitney test): DM-*VEGF*^{KD} - dox vs. +dox (P=0.0823), *VEGF*^{KD} - dox vs. +dox (P=0.6746), *eNOS*$^{-/-}$: *VEGF*^{KD} - dox vs. DM-*VEGF*^{KD} – dox (P=0.0585), *eNOS*$^{-/-}$:*VEGF*^{KD} – dox vs. *VEGF*^{KD} – dox (P=0.6828).
